# CaMKIIδ Splice Variants in the Healthy and Diseased Heart

**DOI:** 10.3389/fcell.2021.644630

**Published:** 2021-03-11

**Authors:** Javier Duran, Lennart Nickel, Manuel Estrada, Johannes Backs, Maarten M. G. van den Hoogenhof

**Affiliations:** ^1^Institute of Experimental Cardiology, Heidelberg University, Heidelberg, Germany; ^2^German Center for Cardiovascular Research (DZHK), Partner Site Heidelberg/Mannheim, Heidelberg, Germany; ^3^Faculty of Medicine, Institute of Biomedical Sciences, University of Chile, Santiago, Chile

**Keywords:** RNA splicing, heart, CaMKII delta, splice variant, therapeutics

## Abstract

RNA splicing has been recognized in recent years as a pivotal player in heart development and disease. The Ca^2+^/calmodulin dependent protein kinase II delta (CaMKIIδ) is a multifunctional Ser/Thr kinase family and generates at least 11 different splice variants through alternative splicing. This enzyme, which belongs to the CaMKII family, is the predominant family member in the heart and functions as a messenger toward adaptive or detrimental signaling in cardiomyocytes. Classically, the nuclear CaMKIIδB and cytoplasmic CaMKIIδC splice variants are described as mediators of arrhythmias, contractile function, Ca^2+^ handling, and gene transcription. Recent findings also put CaMKIIδA and CaMKIIδ9 as cardinal players in the global CaMKII response in the heart. In this review, we discuss and summarize the new insights into CaMKIIδ splice variants and their (proposed) functions, as well as CaMKII-engineered mouse phenotypes and cardiac dysfunction related to CaMKIIδ missplicing. We also discuss RNA splicing factors affecting CaMKII splicing. Finally, we discuss the translational perspective derived from these insights and future directions on CaMKIIδ splicing research in the healthy and diseased heart.

## Introduction

Heart disease is a major cause of death around the world, but the mechanisms underlying its development are not completely understood (Maggioni, 2015). In recent years, (dys)regulation of RNA splicing has been implicated in heart disease, but its contribution to the development and progression of heart disease is not fully known. RNA splicing, a conserved posttranscriptional mechanism, can generally be divided into two processes: constitutive and alternative splicing. In constitutive splicing, which happens in all intron-containing genes and is necessary for the maturation of pre-mRNAs into mRNA, introns are removed from the pre-mRNA and exons are joined together. In alternative splicing, exons of a gene may be included or excluded in the final mature mRNA, producing several different mRNA transcripts from only one gene. More than 95% of mRNAs are subjected to alternative splicing, promoting an increase in the diversity of the transcriptome, which subsequently leads to an increased proteome (Wang et al., 2008). The transcriptome, however, not only includes protein-coding mRNAs, but also non-coding mRNAs that are involved in a multitude of processes, thereby even further increasing the range of functions that are exerted by alternative splicing. Aberrations of alternative splicing can be the cause of multiple diseases including myotonic dystrophy, spinal bulbar atrophy, Prader-Willi syndrome, tauopathies, among others (Tazi et al., 2009).

In the heart, alternative splicing participates in pre- and postnatal development, as well as in the development and progression of heart disease (van den Hoogenhof et al., 2016). The function(s) of multiple splicing factors have been studied by using genetically modified mice, for example by knocking out RNA Binding Motif Protein 20 and 24 (Rbm20 and Rbm24), RNA Binding Fox-1 Homolog 1 and 2 (Rbfox1 and Rbfox2), and the Serine/arginine-rich splicing factor 1 (Srsf1 or Asf/sf2; van den Hoogenhof et al., 2016). Additionally, mutations that impact RNA splicing and induce heart disease have been documented. For example, mutations in splice sites for the genes encoding the Cardiac type Troponin T2 or for Myosin Binding Protein C, which lead to exon skipping or activation of cryptic splice sites, lead to truncated mRNA variants and subsequently impaired sarcomere contractions or hypertrophic cardiomyopathy (Thierfelder et al., 1994; Bonne et al., 1995).

Mutations in splicing factors as a cause for heart disease are only described for RBM20, and these often lead to an arrhythmogenic form of dilated cardiomyopathy (Brauch et al., 2009; Refaat et al., 2012; Parikh et al., 2019). Titin, encoded by the *TTN* gene, is a sarcomeric protein that acts as a scaffolding filament, a signaling platform, and is a principal regulator of contraction in striated muscle. Mutations located in the *RBM20* gene are linked to Titin missplicing toward a larger isoform termed N2BA-Giant, both in animal models and human dilated cardiomyopathy (Guo et al., 2012). In addition to Titin, RBM20 also regulates splicing of other transcripts such as the Ca^2+^ channel ryanodine receptor type 2 (RYR2), the L-type Ca^2+^ channel (LTCC) alpha 1C subunit (CACNA1C/Cav1.2), and the Ca^2+^/calmodulin-dependent protein kinase II delta (CAMK2δ). Missplicing of these genes may contribute to the severity of the cardiomyopathy (Beraldi et al., 2014; Maatz et al., 2014; van den Hoogenhof et al., 2018). In this regard, Rbm20 deficiency induces an intracellular Ca^2+^ overload, similar to what is seen in mice lacking the alternative splice factor Asf/sf2 (Xu et al., 2005; van den Hoogenhof et al., 2018). Interestingly, mice lacking either Rbm20 or Asf/sf2 display a similar splicing switch in Ca^2+^/calmodulin dependent protein kinase II delta (CaMKIIδ) in the heart, from the CaMKIIδB and CaMKIIδC isoform to the CaMKIIδA and CaMKIIδ9 isoform (Xu et al., 2005; Guo et al., 2012; van den Hoogenhof et al., 2018). Traditionally, studies on CaMKII focus on overexpression or activation of CaMKII, but these new findings situate CaMKIIδ splice isoform changes as a focus into the regulators of functional remodeling in the heart.

As an example, mice lacking Asf/sf2 or Rbm20 both missplice CaMKIIδ to the same extent, and it is hypothesized that the switch in CaMKIIδ splice isoforms is one of the causes of Ca^2+^ handling deregulation in these mice (Xu et al., 2005; van den Hoogenhof et al., 2018). Moreover, the functional redundancy of splice factors that alternatively splice CaMKIIδ in the heart, as well as the identification of potential other splice factors deserves additional attention. The (partial) overlap in targets of many cardiac splicing factors, including Asf/sf2 and Rbm20, suggests a coordinated process that needs to be unraveled. Lastly, the functional differences of (increased expression or activation of) the different CaMKIIδ splice variants remain to be investigated. In this review, we will discuss recent discoveries around CaMKIIδ splice variants, how these might control positive and negative changes in the heart, the therapeutic potential derived from these studies, and we will end with potential future directions of CaMKIIδ research.

## Ca^2+^/Calmodulin Dependent Protein Kinases

Ca^2+^/calmodulin-dependent protein kinase (CaMK) belongs to the multifunctional CaMK family. In mammals, CaMKs are divided into 3 classes: CaMKI, CaMKII, and CaMKIV; which in total comprise more than 27 proteins (Swulius and Waxham, 2008; Takemoto-Kimura et al., 2017). The three CaMK classes are expressed in a broad range of cell types, including cardiomyocytes. Activated CaMKI and IV can induce cardiomyocyte hypertrophy, and can activate distinct transcriptional targets in the heart that participate in cardiac remodeling, such as myocyte enhancer factor 2 (MEF2), or synergize with other players involved in cardiac hypertrophy, such as Nuclear factor of activated T cells (NFAT; Passier et al., 2000). In the heart, CaMKII is involved in the control of Ca^2+^ handling and gene transcription, and is suggested as a mediator of mostly (but not only) maladaptive and detrimental effects on cardiac integrity (Bers and Grandi, 2009; Anderson et al., 2011; Beckendorf et al., 2018). Although less is known compared to its function in disease, CaMKII regulates multiple physiological processes such as excitation–contraction coupling (ECC) and excitation–transcription coupling (ETC), the flight or fight response, and contractile force generation (Beckendorf et al., 2018). For example, CaMKII is necessary to increase the heart rate after β-adrenergic stimulation (a.k.a. the fight or flight response). This was first observed in mice with genetic CaMKII inhibition, which surprisingly had a lower heart rate after stress. This could be explained by the effect of CaMKII on the Ca^2+^ uptake and release from the SR in the sinoatrial node (SAN; Wu et al., 2009). Similarly, the LTCC is another important component in the fight or flight response, and this is independent from β-adrenergic stimulation. CaMKII inhibition prevents the increase in heart rate after LTCC stimulation with its agonist BayK, even though *I*_*Cal*_ similarly increased in both control and CaMKII-inhibited SAN cells. This suggests that CaMKII activation is necessary for the heart rate increase in the fight or flight response, also without β-adrenergic stimulation (Gao et al., 2011). In addition to its role in the fight or flight response, CaMKII is also involved in the muscle response to exercise. Multiple studies using CaMKII inhibitors showed the involvement of CaMKII in contractile adaptation and in physiological hypertrophy in response to exercise (Kemi et al., 2007; Burgos et al., 2017). However, these experiments were all performed with complete CaMKII inhibition, and did not focus on specific CaMKII proteins or splice isoforms. Therefore, no conclusions can be made on what CaMKII proteins or splice isoforms are necessary in these processes. However, one could, for example, speculate that the effects on Ca^2+^ handling in the heart during the fight or flight response are likely mediated by CaMKIIδC (see Table 1). In general, even though CaMKII is necessary for some physiological processes, the loss of CaMKII is thought to be protective against heart disease, and sustained activation of CaMKII is therefore proposed to induce maladaptive remodeling.

**TABLE 1:** Phosphorylation targets of CaMKII.

**Phosphorylation target**	**Splice variant**	**References**
PDE4	n.s.	Mika et al., 2015
NOX	n.s.	Pandey et al., 2011
eNOS	n.s.	Murthy et al., 2017
DrP1	n.s.	Bo et al., 2018
McU	n.s.	Joiner et al., 2012
Titin	n.s.	Hidalgo et al., 2013
MyBP-C	n.s.	Hartzell and Glass, 1984
NHE	n.s.	Vila-Petroff et al., 2010
LTCC	n.s.	Anderson et al., 1994; Koval et al., 2010
RyR	CaMKIIδC, but not CaMKIIδB	Zhang et al., 2007; Di Carlo et al., 2014
PLN	CaMKIIδC, but not CaMKIIδB	Zhang et al., 2003
Calcineurin	n.s.	Kreusser et al., 2014
HDAC	CaMKIIδA, CaMKIIδB, CaMKIIδC	Backs et al., 2006; Zhang et al., 2007
UBE2T	CaMKIIδ9, but not CaMKIIδA or CaMKIIδB	Zhang et al., 2019
HSF1	CaMKIIδB	Holmberg et al., 2001; Peng et al., 2010
CREB	n.s.	Sun et al., 1994
NF-κB	CaMKIIδC, but not CaMKIIδB	Gray et al., 2017
IKK	n.s.	Martin et al., 2018
Histone 3	CaMKIIδB	Awad et al., 2013
Different ion channels		See review Hegyi et al., 2019

*List of known CaMKII phosphorylation targets, and which CaMKII splice isoforms are able to phosphorylate these targets.n.s., not specified.*

## Ca^2+^/Calmodulin Dependent Protein Kinase Ii

**FIGURE 1:**
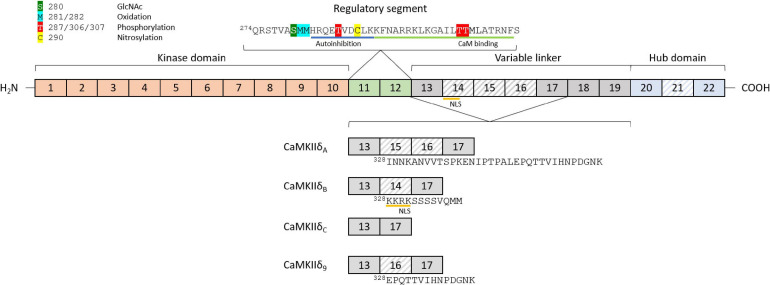
Structure and features of the *CaMKII*δ gene and exon composition of different splice isoforms. The basic structure of the *CaMKII*δ gene consists of a kinase domain (exons 1–10), a regulatory segment (exons 11–12), a variable linker (exons 13–19), and a hub domain (exons 20–22). The variable regions in different splice isoforms are located between exons 13 and 17 (variable domain 1) and exons 20 and 22 (variable domain 2). Different posttranslational modifications can lead to activation or inactivation of CaMKIIδ, including phosphorylation, oxidation, nitrosylation, and *O*-linked *N*-acetylglucosamine. CaM-binding and autoinhibition regions are marked in the regulatory segment. The four major cardiac splice isoforms are shown with their different exon compositions in the variable region 1. Differentially spliced exons are striped, and amino acid sequences of the differently spliced region are added starting at position 328.

Ca^2+^/calmodulin dependent protein kinase II proteins are encoded by four separate genes, *CaMKII*α/β/γ/δ, each one with a different expression pattern. CaMKIIα and CaMKIIβ are mainly expressed in the brain and mediate synaptic functions underlying learning, memory, and cognition (Cook et al., 2018). Their expression in the heart is still under debate, since some authors suggest that the α and β genes are not detectable in the heart (Kreusser et al., 2014), while others report the presence of CaMKIIα and β in ventricular cardiomyocytes (Cipolletta et al., 2015). CaMKIIγ and CaMKIIδ are expressed and found in the healthy and diseased heart, with CaMKIIδ being the highest expressed, and only these CaMKII genes have been knocked out specifically in the heart (Edman and Schulman, 1994; Colomer et al., 2003; Kreusser et al., 2014). The basic structure of CaMKIIδ consists of a specific Ser/Thr kinase domain at the *N*-terminus (exons 1–10), a regulatory segment (exons 11–12), a variable linker (exons 13–19), and a hub domain (exons 20–22; Figure 1). Under basal conditions, the kinase is in an autoinhibited state, with the regulatory domain acting like a substrate for the catalytic domain (Kelly et al., 1988; Hoffman et al., 2011). Binding of calmodulin induces a conformational change, which releases the association of the regulatory and catalytic domain, rendering the enzyme active (Kolodziej et al., 2000; Gaertner et al., 2004; Myers et al., 2017). Under physiological conditions, CaMKIIδ activity is typically driven by the presence of Ca^2+^/CaM. However, posttranslational modifications can result in the prevention of reassociation between the regulatory and catalytic domains, thereby converting the enzyme to a persistently active state. These posttranslational modifications include autophosphorylation at Thr-287 (Lai et al., 1987) and Thr-306/307 (Patton et al., 1990; Lu et al., 2003), oxidation at Met-281/282 (Erickson et al., 2008), and Met-308 (Konstantinidis et al., 2020), O-GlcNAc modification at Ser-280 (Erickson et al., 2013), and nitrosylation at Cys-290 (Erickson et al., 2015; Figure 1). While posttranslational modifications in between the catalytic domain and the CaM binding site lead to autonomous activity, modifications within the CaM binding site, in contrast, appear to have an inhibitory effect. For example, phosphorylation of Thr-306/307 has an inhibitory effect on CaMKII activity, as this modification prevents binding of CaM (Lu et al., 2003). CaMKII proteins can form large oligomeric structures of 12 subunits from one or a combination of different isoforms, with the carboxy-terminal hub domains centrally located and the kinase domains arranged in a circle around the hub center, connected by the linker region. Oligomerization is necessary for rapid inter-subunit autophosphorylation at Thr-287 (Thr-286 in CaMKIIα) by catalytic domains of other subunits localized in the same oligomer during longer periods of Ca^2+^/CaM binding (Lai et al., 1987). In the phosphorylated state, the affinity of Ca^2+^/CaM binding is increased while the release is slowed down, a process known as Calmodulin trapping (Meyer et al., 1992). This enables the activity of CaMKII even with decreasing Ca^2+^ concentrations, until the phosphate group is removed by a phosphatase (Strack et al., 1997). Oxidation of CaMKIIδ at Met-281/282 works as a sensor system for reactive oxygen species (ROS) and therefore oxidative stress in the heart. Altered oxidation has been shown in a variety of cardiac disease models and can lead to arrhythmias, suggesting that CaMKIIδ-specific antioxidants could serve as a potential future therapeutic agent (Luczak and Anderson, 2014). Nitrosylation also appears to be an important modification of key elements of cardiac function, including CaMKIIδ. *S*-nitrosylation of Cys-290 site leads to sustained autonomous CaMKIIδ activation, whereas *S*-nitrosylation at Cys-273 inhibits CaMKIIδ activation when NO donors are present before Ca^2+^/CaM is available (Erickson et al., 2015). The existing dual mechanisms of activating and deactivating modifications of the same nature (phosphorylation and nitrosylation) suggest that the regulation of CaMKIIδ activity by posttranslational modifications is much more complex than previously assumed. Especially the dynamics of the presence or absence of different modifications in relation to varying Ca^2+^/CaM concentrations should be the aim of future experiments. Ser-280 can be O-GlcNAcylated in the presence of elevated glucose concentrations, leading to activation of CaMKIIδ (Erickson et al., 2013). The enzyme *O*-GlcNAc transferase catalyzes this modification in the presence of UDP-*N*-acetylglucosamine, which is formed as a product of the of the hexosamine biosynthetic pathway (Hart and Akimoto, 2009). This modification is particularly important in the diabetic heart, where the ratio of *O*-GlcNAc-modified CaMKII to total CaMKII is increased due to changes in glucose signaling (Erickson et al., 2013; Lu et al., 2020). Whether alternative splicing increases the susceptibility of specific splice isoforms to these PTMs, or whether alternative splicing induces new potential PTM sites is currently unknown.

Ca^2+^/calmodulin dependent protein kinase II gamma, the second most expressed CaMKII protein in the heart, has 4 described splice variants (Singer et al., 1997). However, the relationship between CaMKIIγ and cardiovascular disease is poorly explored. In this regard, CaMKIIγ is involved in atherosclerotic plaque development, which could drive myocardial infarction (Doran et al., 2017), and its mRNA levels are increased in pressure overload-induced cardiac hypertrophy in mice and in Rbm20-deficient rats (Guo et al., 2012; Kreusser et al., 2014). Cardiomyocyte specific deletion of CaMKIIγ shows a decrease in cardiomyocyte apoptosis and a reduction of cardiac hypertrophy induced by transverse aortic constriction and isoproterenol treatment in mice (Kreusser et al., 2014).

**FIGURE 2:**
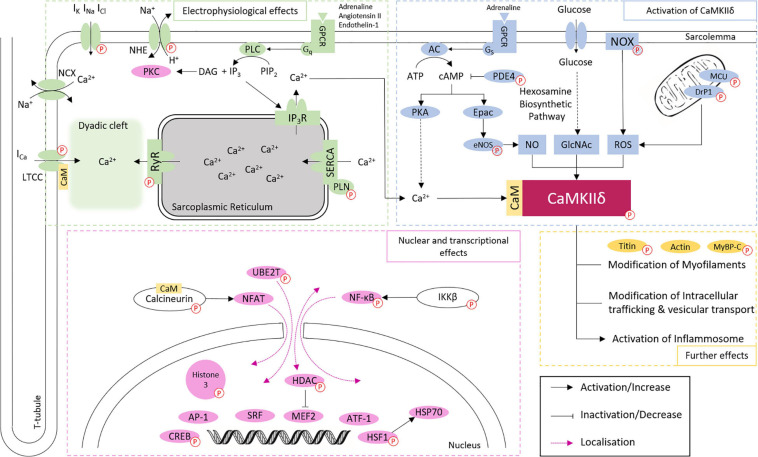
Overview of CaMKIIδ activation and downstream signaling in cardiomyocytes. CaMKIIδ can be activated via Ca^2+^/CaM binding, autophosphorylation, and different posttranslational modifications. The phosphorylation of different proteins (indicated by *P* symbol) by CaMKIIδ can have both activating and inactivating functions. This affects cardiac electrophysiology, especially Ca^2+^ handling, gene expression, intracellular trafficking, mitochondrial processes, energetics, cell-cell coupling, inflammatory reactions, and the cytoskeletal network. Different phosphorylation targets are listed with a corresponding reference in Table 1. Targets linked to phosphorylation by CaMKIIδ, but without proven direct phosphorylation, are indicated by striped *P* symbols.

Ca^2+^/calmodulin dependent protein kinase II delta is best known to participate in the regulation of Ca^2+^ handling (Zhang et al., 2007), through the phosphorylation of the LTCC, which facilitates intracellular Ca^2+^ entry (Koval et al., 2010), the sarcoplasmic reticulum (SR) membrane protein Phospholamban (PLN), with the consequent increase in Ca^2+^ uptake from cytoplasm into SR lumen by the Sarco/endoplasmic reticulum Ca^2+^-ATPase (SERCA2a; Mattiazzi and Kranias, 2014), and RyR2 which increases SR Ca^2+^ leak into the cytoplasm (Witcher et al., 1991; van Oort et al., 2010). Additionally, CaMKIIδ regulates gene transcription, e.g., through phosphorylation of histone deacetylase 4 (HDAC4; Backs et al., 2006, 2009) and Histone 3 (Awad et al., 2013; Saadatmand et al., 2019), mitochondrial reprogramming (Westenbrink et al., 2015), and inflammasome activation (Suetomi et al., 2018; Willeford et al., 2018). These effects are closely related to each other and are involved in the development of physiological and pathophysiological effects of CaMKIIδ in the heart (Figure 2 and Table 1). However, it cannot be ruled out that other CaMKIIδ functions, independent of calcium handling and transcriptional regulation, contribute significantly to heart disease since many other phosphorylation targets of CaMKII have been described.

Both global and cardiomyocyte specific deletion of CaMKIIδ protects against adverse cardiac remodeling (Backs et al., 2009; Ling et al., 2009, 2013), while transgenic overexpression of CaMKIIδ promotes cardiac hypertrophy or dilated cardiomyopathy (Zhang et al., 2002, 2003, 2019; Xu et al., 2005). Similar to CaMKIIγ deletion in the heart, CaMKIIδ cardiomyocyte knockout (KO) mice show an only partial decrease in known target phosphorylation, and reduction of cardiac hypertrophy in mice subjected to transverse aortic constriction surgery. While this is less evident in some CaMKIIδ KO mouse models (Ling et al., 2009) than in others (Kreusser et al., 2014), the data suggests redundancy of these two genes.

Cardiomyocyte specific deletion of CaMKIIδ attenuates the increase in the inflammatory mediators CCL2/CCL3, NFkB and its downstream targets IL-1β, IL-6, CXCL1, TNF-α, and NLRP3 in non-ischemic and ischemic cardiac disease (Weinreuter et al., 2014; Suetomi et al., 2018; Willeford et al., 2018). In line with this, hearts from cardiomyocyte specific CaMKIIδ KO mice demonstrate that cardiac infarct formation in ischemia/reperfusion injury, as well as the upregulation of the NFκB and gene expression of pro-inflammatory TNF-α and IL-6 are dependent on CaMKIIδ specifically in cardiomyocytes (Ling et al., 2013; Gray et al., 2017). Due to the redundant contribution of CaMKIIγ and CaMKIIδ in cardiomyocyte apoptosis and cardiac hypertrophy, the generation of a double KO (DKO) for CaMKIIγ and CaMKIIδ was necessary to clarify the role of CaMKII in the heart. DKO mice show that both genes contribute redundantly to phosphorylation of PLN (Thr-17), RyR2 (Ser-2814), HDAC4 (Ser-632), and calcineurin (Ser-411). Moreover, DKO mice show protection against cardiac dysfunction and interstitial fibrosis induced by pressure overload and chronic β-adrenergic stimulation (Kreusser et al., 2014). These mice, similar to CaMKIIδ KO generated by another group (Ling et al., 2013), are protected against post-infarct remodeling and inflammatory processes in the heart (Weinreuter et al., 2014). Unexpectedly, the deletion of both genes does not inhibit cardiac hypertrophy, due the hypo-phosphorylation of calcineurin, which in turn increases calcineurin activity (Kreusser et al., 2014). This uncovered CaMKIIδ to be not only a maladaptive effector in the heart, but also a regulator of physiological calcineurin-induced cardiac hypertrophy.

In short, CaMKIIδ is a central transducer of intra- and extra-cellular signaling in physiological processes, but is also involved in a multitude of maladaptive processes (Figure 2 and Table 1). The question arises how a protein can have this degree of diversity in its function. We hypothesize that this is, at least in part, due to the different splice isoforms of CaMKIIδ.

## CaMKIIδ Splice Variants

The first description of different splice isoforms of CaMKIIδ was made by Schworer et al. (1993), after which additional splice isoforms were discovered and described in different tissues (Edman and Schulman, 1994; Srinivasan et al., 1994) [for an overview see Beckendorf et al. (2018)]. Until now, 11 different splice isoforms have been described, but not all of them are expressed in the heart (Zhang et al., 2019). The isoforms differ in inclusion of exons between exons 13–17, or exons 20–22 (Figure 1). Initially, research focus lay on the CaMKIIδC (a.k.a. CaMKIIδ2) and CaMKIIδB (a.k.a. CaMKIIδ3) splice isoforms in the heart. Only later CaMKIIδA, and very recently CaMKIIδ9 were described as mediators in the pathogenesis of cardiac disease (Xu et al., 2005; Zhang et al., 2019).

Ca^2+^/calmodulin dependent protein kinase heteromultimerization can include different CaMKII proteins and splice isoforms from those proteins. The ratio of CaMKII proteins and splice isoforms can regulate the localization of the holoenzyme (Mishra et al., 2011). Thus, it is hypothesized that the switch in CaMKIIδ isoform ratio might determine the effect on phosphorylation sites in proteins present in different subcellular locations, e.g., high relative expression of nuclear CaMKIIδB might phosphorylate proteins involved in gene transcription, such as HDAC4/5, and high relative expression of cytoplasmatic CaMKIIδC on Ca^2+^-handling proteins, such as PLN or RyR2.

The RNA binding proteins Rbfox1 and Rbfox2, Asf/sf2, Sc35, and Rbm20 can regulate CaMKIIδ splicing, suggesting that CaMKIIδ splicing is a dynamic and regulated process, which should be explored extensively (Gray and Heller Brown, 2014; Beckendorf et al., 2018). The first study that showed a shift in CaMKIIδ splicing, from the CaMKIIδB/δC isoforms toward CaMKIIδA, characterized the cardiomyocyte specific KO of the splicing factor Asf/sf2. These mice presented with cardiomyopathy and cardiomyocyte Ca^2+^ handling disturbances, but this phenotype was not seen in the cardiomyocyte specific KO of the Asf/sf2-related splicing regulator Sc35 (Xu et al., 2005). *In vitro*, Sc35 has been described as a CaMKIIδ splicing regulator, but its *in vivo* relevance is not entirely clear (Han et al., 2011). Cardiac specific KO of Rbfox1 or Rbfox2 induces CaMKIIδ missplicing and these mice present with cardiomyopathy. However, this phenotype does not seem to depend on CaMKIIδ missplicing, at least in the Rbfox1 KO, as re-expression of proper Mef2 isoforms rescues the phenotype (Wei et al., 2015; Gao et al., 2016). The last known CaMKIIδ splicing regulator is Rbm20, of which KO leads to increased expression of CaMKIIδA and CaMKIIδ9, and disturbed intracellular Ca^2+^-handling, similar to what is seen in cardiomyocyte specific Asf/sf2 KO mice (van den Hoogenhof et al., 2018).

Patients with mutations in RBM20 are at high risk of suffering from sustained ventricular arrhythmias, which might be related to changes in Ca^2+^ handling due to CaMKIIδ misplicing into CaMKIIδA and CaMKIIδ9 (van den Hoogenhof et al., 2018). Increased CaMKIIδA expression is associated with enhanced L-type Ca^2+^ current density (Xu et al., 2005; Koval et al., 2010). Later on, we will discuss the discoveries related to this isoform, but it illustrates the relevance to understand the functional consequences of different CaMKIIδ splice isoforms.

## CaMKIIδB

Ca^2+^/calmodulin dependent protein kinase II deltaB, characterized traditionally as the nuclear CaMKIIδ splice isoform, is together with CaMKIIδC the most studied CaMKIIδ splice isoform in the heart. It is the only CaMKIIδ splice isoform comprising exon 14, which is translated in a 11 amino acid insert. The first four amino acids of this exon correspond to the nuclear localization signal ^332^KKRK, which locates CaMKIIδ to the nucleus (Figure 2; Ramirez et al., 1997). However, phosphorylation of CaMKIIδB on Thr-287 curiously localizes the protein in the cytoplasm, but this can be overcome by changing Ser-332 (immediately adjacent to the NLS) to a phosphoresistant Ala, which restores nuclear localization of CaMKIIδB (Backs et al., 2006). In addition, CaMKIIδB can be phosphorylated at this residue, possibly by CaMKI or CaMKIV, which induces nucleocytoplasmic shuttling and limits its nuclear localization (Heist et al., 1998; Backs et al., 2006).

Similar to other splice isoforms, CaMKIIδB expression varies in cardiac development and remodeling. For example, in cultured cardiac progenitor cells CaMKIIδB is upregulated during differentiation and compartmentalizes mostly in the nucleus, after which CaMKIIδB expression decreases in adulthood (Quijada et al., 2015). Interestingly, CaMKIIδB overexpression in cardiac progenitor cells reduces proliferation rate and increases cell size, which suggests that CaMKIIδB drives cardiac progenitor cell commitment (Quijada et al., 2015). However, *in vivo* lineage tracing experiments are required to further clarify the role of CaMKIIδB in this process. Furthermore, CaMKIIδB is upregulated in mouse hearts after myocardial infarction, and in failing human hearts (Hoch et al., 1999; Little et al., 2009; Quijada et al., 2015). In doxorubicin-induced cardiomyopathy and Rbm20 cardiomyopathy, on the other hand, CaMKIIδB is downregulated (Little et al., 2009; van den Hoogenhof et al., 2018). These studies show the versatility in CaMKIIδB expression in different stages in cardiomyocytes.

Because of its subcellular localization, CaMKIIδB has been linked to gene transcription regulation. In cardiomyocytes, CaMKIIδB overexpression in cardiomyocytes increases the activity of an atrial natriuretic factor (*ANP*) gene reporter as well as Anf protein expression (Ramirez et al., 1997). Interestingly, the use of chimera proteins of different CaMKII splice isoforms with the NLS encoded in exon 14 (which is normally only included in CaMKIIδB) similarly increased activity of the ANP gene reporter, suggesting that nuclear localization is sufficient to drive gene expression downstream of CaMKII activation (Ramirez et al., 1997). Furthermore, CaMKIIδB can phosphorylate nuclear epigenetic targets such as HDAC4 and HDAC5 and Histone 3 (at Ser-10 and Ser-28; Backs et al., 2006; Saadatmand et al., 2019). Phosphorylation of HDAC4 and HDAC5 increases their nuclear export, and is related with activation of a hypertrophic program downstream of MEF2 and GATA4 (Lu et al., 2010; Awad et al., 2013, 2015). Increased phosphorylation of Histone 3 is correlated with increased hemoglobin expression, but the functional relevance of this event is not yet known (Saadatmand et al., 2019). However, the premise that hemoglobin is not only expressed in cardiomyocytes, but also has a function in these cells is intriguing and deserves additional attention.

Multiple works also showed the protective effect of CaMKIIδB (Little et al., 2009; Peng et al., 2010). For example, overexpression of CaMKIIδB in primary neonatal cardiomyocytes suppresses apoptosis through increased expression of the anti-apoptotic modulator B-cell lymphoma 2 (Bcl-2) and phosphorylation and activation of HSF1 (Peng et al., 2010). Similarly, overexpression of CaMKIIδB in cardiac progenitor cells protects from oxidative stress damage, while knocking down CaMKIIδB increases cell death and expression of senescence markers (Quijada et al., 2015). In rats with Doxorubicin-induced cardiomyopathy, CaMKIIδB expression decreases, and together this suggests that CaMKIIδB is necessary to limit cardiac cell death (Little et al., 2009). It would be interesting to restore CaMKIIδB expression in this model, e.g., though AAV-mediated gene transfer, and see if this is protective. In line with this, CaMKIIδ-KO mice are protected from cardiac dysfunction in an ischemia/reperfusion injury model in mice, and transgenic CaMKIIδB overexpression in the CaMKIIδ-KO further protects by inhibiting the upregulation of pro-inflammatory cytokines and decreasing infarct size (Gray et al., 2017). Whether CaMKIIδB is also involved in the regulation of the inflammatory response after non-ischemic stress remains to be seen, but this is clinically interesting as cardiac inflammation commonly occurs in heart failure patients (Elster et al., 1956; Suetomi et al., 2019; Adamo et al., 2020). CaMKIIδB overexpression is not only protective in ischemic stress, but also in cultured cardiomyocytes exposed to Angiotensin II. CaMKIIδB phosphorylates the transcription factor HSF1, and this increases the expression of its downstream target gene inducible (i)HSP70, which in turn suppresses stress-induced apoptotic signals of multiple Bcl-2 members and therefore acts as an anti-apoptotic chaperone (Peng et al., 2010).

Cardiomyocyte specific transgenic overexpression of CaMKIIδB *in vivo* promotes the development of cardiac hypertrophy at 4 months of age, even without an experimental intervention (Zhang et al., 2002). However, even though CaMKII activity is increased in these hearts, CaMKII-dependent phosphorylation of PLN (Thr-17) and RyR2 (Ser-2814) was not, and cardiac Ca^2+^ handling was equally not affected (Zhang et al., 2002, 2007). However, transgenic CaMKIIδB overexpression does increase HDAC4 phosphorylation, which shuttles HDAC4 out of the nucleus. Subsequently, MEF2 is derepressed, and transcription of pro-hypertrophic genes, such as Anf, Bnp, β-mhc and skeletal actin is increased (Backs et al., 2006; Little et al., 2007; Zhang et al., 2007). Summarizing, CaMKIIδB can be both detrimental and protective, depending on what downstream processes are activated. On the one hand, CaMKIIδB can activate pro-hypertrophic and detrimental HDAC4-MEF2 signaling, and CaMKIIδB transgenic mice have a, albeit relatively mild, cardiac phenotype (Zhang et al., 2002; See Table 2). On the other hand, CaMKIIδB expression protects against *in vivo* ischemic injury (Gray et al., 2017). Further research is needed to clarify when CaMKIIδB expression is protective, and when it is not. Additionally, the simultaneous activation of detrimental transcription factors such as MEF2 and protective transcription factors such as HSF1 suggests that transcription factors downstream CaMKIIδB do not function in only one direction, and that their interaction should be further explored.

**TABLE 2:** Overview of *in vivo* studies of CaMKIIδ splice variant overexpression in cardiomyocytes.

**Splicevariant**	**Mouse model**	**Intervention/Cardiac Phenotype**	**Ca^2+^ handling/Major signaling**	**References**
**CaMKIIδA**	Cardiomyocyte specific transgenic overexpression (αMHC-driven)	• Cardiac hypertrophy at 4 weeks of age • Contraction defects • Death at 8 weeks of age	• Ca^2+^ handling defects	Xu et al., 2005
	Cardiomyocyte specific transgenic overexpression (αMHC-driven)	• Activation of hypertrophic gene program at 4 weeks of age • Cardiac hypertrophy and moderate cardiac dysfunction at 4 months of age	• Downregulation of PLN phosphorylation (Ser16/Thr17) • Decrease in SR Ca^2+^ uptake (*in vitro*) • Repression of HDAC4 and upregulation of MEF2 activity • Increase in PP2A activity	Zhang et al., 2002, 2007
**CaMKIIδB**	AAV9-mediated overexpression in cardiomyocytes in CaMKIIδ/γ (αMHC-driven) KO background	• Protection against myocardial damage and dysfunction following 1 hour *ex vivo*I/R	-	Weinreuter et al., 2014
	Cardiomyocyte specific transgenic overexpression (αMHC-driven) in cardiomyocytes (αMHC-driven) CaMKIIδ KO background	• Cardiac damage not affected after I/R injury for 1 day	• Upregulation of IL-6 mRNA	Gray et al., 2017
	Cardiomyocyte specific transgenic (αMHC-driven)	• Cardiac hypertrophy at 6 weeks of age • Heart failure at 12 weeks of age	• Ca^2+^ handling defects • Repression of HDAC4 and upregulation of MEF2 activity	Maier et al., 2003; Zhang et al., 2003, 2007; Ljubojevic-Holzer et al., 2020
	AAV9-mediated overexpression in cardiomyocytes in CaMKIIδ/γ (αMHC-driven) KO background	• Cardiac damage not affected after I/R injury for 1 day	-	Weinreuter et al., 2014
**CaMKIIδC**	Cardiomyocyte specific transgenic overexpression (αMHC-driven) in cardiomyocyte (αMHC-driven) CaMKIIδ KO background	• Exacerbated myocardial damage and dysfunction following 1 hour *ex vivo*I/R	• Upregulation of IKK-α/β phosphorylation and NF-κB protein content • Upregulation of TNF-α and IL-6 mRNA	Gray et al., 2017
	Cardiomyocyte specific transgenic (αMHC-driven) plus mitochondrial localization signal	• Modest cardiac hypertrophy (Without cardiomyocyte cross-sectional area changes) • Dilated cardiomyopathy	• MCU phosphorylation • Decrease in ATP content • Decreased mitochondrial complex I and increased complex II activity	Luczak et al., 2020
**CaMKIIδ9**	Cardiomyocyte specific transgenic (αMHC-driven)	• Cardiac hypertrophy at 6 weeks of age • Heart failure at 10 weeks of age	• Disruption of UBE2T-dependent DNA repair pathway • DNA damage	Zhang et al., 2019

## CaMKIIδC

Classically known as the cytoplasmic CaMKIIδ isoform, CaMKIIδC lacks exons 14–16, and is therefore the smallest CaMKIIδ splice variant (Figure 2; Schworer et al., 1993). Although initial studies described this isoform exclusively in the cytoplasm (Edman and Schulman, 1994), in cardiomyocytes it is also associated with the SR membrane, the plasma membrane, and the nuclear membrane (Mishra et al., 2011; Ljubojevic-Holzer et al., 2020), where it possible translocates together with CaMKIIδB (Mishra et al., 2011). Therefore, even though this splice isoform lacks the NLS, it is nevertheless found in the nuclear compartment, which most likely relates to the composition of the holoenzyme, which includes (different ratios) of all CaMKII proteins and isoforms (Mishra et al., 2011). Together with CaMKIIδB, CaMKIIδC was initially thought to be the most expressed isoform in the heart, but recent results challenge this view and situate CaMKIIδC as only the third most highly expressed isoform in the human, rhesus monkey and rat heart (Zhang et al., 2019). CaMKIIδC expression increases in the postnatal heart after day 2 (Quijada et al., 2015), in mice exposed to TAC surgery (Zhang et al., 2003), and in patients with heart failure (Ljubojevic-Holzer et al., 2020).

The first study with CaMKIIδC overexpression showed that adenoviral CaMKIIδC overexpression in adult cardiomyocytes increases activation of β1-adrenergic-induced apoptosis (Zhu et al., 2003, 2007). Moreover, inhibition of CaMKII activity through the use of KN-93 or AIP, or the use of a dominant negative mutant of CaMKIIδC, blocks the increase in apoptosis induced by both the overexpression of CaMKIIδC or cell death stimuli (Zhu et al., 2007). CaMKIIδC also upregulates the proapoptotic transcription factor p53 *in vivo*, which in part could explain the effects of CaMKIIδC in cardiomyocyte apoptosis (Toko et al., 2010). Interestingly, these data suggest that CaMKIIδC and CaMKIIδB have opposite roles when it comes to apoptosis, as CaMKIIδC induces apoptosis while CaMKIIδB protects against apoptosis.

Cardiomyocyte specific overexpression of CaMKIIδC in mice leads to rapidly progressing cardiac hypertrophy that transitions to heart failure at 3 months of age (see Table 2), as well as a dysregulation of Ca^2+^ handling. This is accompanied by an increase in PLN (Thr-17) and RyR2 (Ser-2814) phosphorylation (Zhang et al., 2003), and a reduction in total protein of PLN, RyR2, SERCA2, and upregulation of the Na^+^-Ca^2+^ exchanger (Maier et al., 2003). Even though increased PLN phosphorylation should increase SERCA function, this is offset by the decrease in SERCA and PLN protein levels. Therefore, decreased SERCA function and increased NCX function leads to a lower SR Ca^2+^ content and Ca^2+^ transient amplitude, demonstrating the involvement of CaMKIIδC in ECC regulation (Maier et al., 2003). Interestingly, cardiomyocyte specific overexpression of CaMKIIδC in a CaMKIIδ-KO background induces a more severe phenotype than overexpression of CaMKIIδC in a wildtype background (Ljubojevic-Holzer et al., 2020). This suggests that (the ratio of) different CaMKIIδ splice isoforms may modulate long-term dysfunction induced by CaMKIIδC overexpression in cardiomyocytes.

The detrimental effects of CaMKIIδC activation seems to depend on timing as well. Early activation of CaMKIIδC after TAC (i.e., 5 days after TAC) increases both SR Ca^2+^ content and release, possibly to compensate the afterload imposed by TAC (Ljubojevic-Holzer et al., 2020). Notably, in mice subjected to acute pressure overload, pharmacological inhibition of CaMKII shows an impairment of ECC and decreased survival, suggesting that inhibiting the early CaMKII response can be detrimental (Baier et al., 2020). These early adaptive effects are also seen in CaMKIIδC transgenic mice. For example, young mice overexpressing CaMKIIδC (6–8 weeks) show similar compensatory Ca^2+^ transient effects and do not present with cardiac dysfunction (Zhang et al., 2003; Ljubojevic-Holzer et al., 2020). However, over time these compensatory effects are reversed, both after TAC and in CaMKIIδC TG mice, and these mice progress to HF (at 45 days and 11–13 weeks, respectively; Ljubojevic-Holzer et al., 2020). This suggests that CaMKIIδC is necessary for an early adaptive response, but detrimental at later stages. It raises the questions whether CaMKIIδ splicing itself is affected differently in different disease stages, and if it could be beneficial to redirect splicing toward isoforms that are specifically needed during different phases of disease (e.g., increased expression of CaMKIIδC in the early stage of disease).

Crossing CaMKIIδC transgenic mice with PLN KO mice (CaMKIIδC-TG/PLN-KO) attenuates the Ca^2+^ handling defects seen in transgenic mice overexpressing CaMKIIδC, which is in line with the hypothesis that the decreased SR Ca^2+^ uptake through SERCA underlies the Ca^2+^ handling defects. However, while the SR Ca^2+^content and Ca^2+^ transient amplitude were normalized, left ventricular dilation, ventricular function, apoptosis, and mortality were exacerbated (Zhang et al., 2010). This can be explained in part by the increased phosphorylation of RyR2, which together with the restored SR Ca^2+^content, increased the SR Ca^2+^ spark frequency and SR Ca^2+^ leak. Interestingly, inhibiting SR Ca^2+^ leak through the use of Ryanodine in CaMKIIδC-TG/PLN-KO cardiomyocytes improved the viability and prevented the increase in apoptosis (Zhang et al., 2010). On the other hand, CaMKIIδC transgenic mice crossed with mice expressing a SR-targeted autocamtide-2-related inhibitory peptide (SR-AIP) show a reduction in PLN and RyR2 phosphorylation, but no improvement in cardiac function, suggesting that the cardiac dysfunction in CaMKIIδC transgenic mice is not only dependent on phosphorylation of its SR-associated targets (Huke et al., 2011).

These effects on Ca^2+^ handling are distinct from the effects of CaMKIIδB overexpression, but overexpression of both splice variants similarly leads to HDAC4 phosphorylation (Ser-632), which inhibits nuclear import, and subsequently induces MEF2 transcriptional activity (Backs et al., 2006; Zhang et al., 2007). Additionally, Ljubojevic-Holzer et al. demonstrated recently that in mice subjected to TAC surgery and in failing human hearts the perinuclear and nuclear CaMKII population correspond majorly to CaMKIIδC, which suggests that CaMKIIδC also regulates gene expression in cardiac remodeling (Ljubojevic-Holzer et al., 2020). CaMKII phosphorylates class IIa HDACs, but overexpression of CaMKIIδC also affects class I HDAC activity (Zhang et al., 2020). CaMKIIδC increases HDAC1 activity, and mice overexpressing CaMKIIδC show an increase in HDAC1 and HDAC3 expression. Interestingly, class I HDAC inhibition attenuates hypertrophy and maladaptive remodeling in CaMKIIδC transgenic mice, suggesting that, at least in part, the pro-hypertrophic effect of increased CaMKIIδC expression is dependent on class I HDAC activity (Zhang et al., 2020). Opposite to the protective effect of CaMKIIδB in I/R in mice, CaMKIIδC overexpression in a CaMKIIδ-KO background exacerbates cardiac dysfunction and increases infarct size. This effect is mediated by increased phosphorylation of IKK and nuclear localization of NF-κB, which is accompanied by an upregulation of TNFα and IL-6 in *ex vivo* hearts exposed to I/R (Gray et al., 2017). This suggests that CaMKIIδ mediates inflammasome activation and macrophage recruitment in non-ischemic injury through a transcriptional mechanism involving NF-κB, which is supported by multiple other studies (Weinreuter et al., 2014; Suetomi et al., 2018; Willeford et al., 2018). Thus, NF-κB activation represents another transcriptional pathway through which CaMKIIδ exerts its pathophysiological effects on the heart. However, while it is shown that CaMKIIδB and CaMKIIδC have opposite effects in this regard, the specific action of different CaMKIIδ splice isoforms is not entirely understood.

Ca^2+^/calmodulin dependent protein kinase II is also implicated in the regulation of mitochondrial processes (Odagiri et al., 2009). After the observation that mitochondrial Ca^2+^ and apoptosis are elevated in mice overexpressing CaMKIIδC (Zhu et al., 2007, Zhang et al., 2010), the Anderson group demonstrated that mice with specific mitochondrial CaMKII inhibition are resistant to I/R injury and myocardial infarction (Joiner et al., 2012). Mitochondrial CaMKII inhibition decreased phosphorylation of the inner membrane mitochondrial Ca^2+^ uniporter (MCU), which is the transmembrane protein that allows the passage of Ca^2+^ from cytoplasm into the inner mitochondria (Joiner et al., 2012). The phosphorylation of MCU by CaMKII increases *I_*MCU*_*, which in turn promotes mitochondrial permeability transition pore (mPTP) opening, triggering programmed cell death (Joiner et al., 2012; Halestrap and Richardson, 2015). However, this result is in discrepancy with a study from the Kirichok group, which using similar conditions, reported that *I*_*MCU*_ in cardiomyocytes is very small and is not directly regulated by CaMKII (Fieni et al., 2012, 2014). These studies were performed in mitoplasts with perfusion of constitutively active CaMKII, and the contribution of CaMKII in mitochondria *in vivo* will clarify whether the specific action of this enzyme on *I*_*MCU*_ underlies the protective effect of mitochondrial CaMKII inhibition in the prevention of cardiac dysfunction. Another recent study reports that CaMKIIγ/δ deletion does not affect mitochondrial *I*_*MCU*_ upon β-adrenergic or electrical stimulation in isolated cardiomyocytes, nor in cardiomyocyte-isolated mitochondria subjected to oxidative stress (Nickel et al., 2020). Mitochondria-targeted CaMKIIδC overexpression causes dilated cardiomyopathy, but with modest cardiac hypertrophy and no changes in cardiomyocyte cross-sectional area or cell death. It also reduces expression of assembled complex I, the mitochondrial isoform of creatine kinase and increases the production of NADH (Luczak et al., 2020). However, the finding that CaMKIIγ/δ deletion does not change redox state of mitochondria, measured by NADH accumulation, argues that while CaMKIIδC seems sufficient to increase NADH production, CaMKIIγ/δ as a whole is not necessary (Nickel et al., 2020). Another question that remains is related with the equal or differential action of cytoplasmic and mitochondrial CaMKII pools. Independent groups demonstrated the upregulation of CaMKIIδC in the mitochondrial fraction derived from hearts exposed to ischemia/reperfusion and the involvement of CaMKIIδC in mitochondrial Ca^2+^ content (Zhang et al., 2010; Weinreuter et al., 2014). In addition, in transgenic mice with cardiomyocyte sustained activation of Gαq signaling, which present with mitochondrial dysfunction and upregulation of mitochondrial ROS, the additional cardiomyocyte-specific deletion of CaMKIIδ attenuates these changes in mitochondrial function (Westenbrink et al., 2015). This suggests the involvement of CaMKIIδ in mitochondrial (dys)function upon sustained Gαq signaling. Overall, it is clear that both cytoplasmic and mitochondrial CaMKII pools can affect mitochondrial function, but the underlying mechanisms, and the specific actions of different CaMKIIδ isoforms, remain to be investigated. In conclusion, CaMKIIδC is involved in a multitude of processes in the development of heart failure and arrhythmias (Gray and Heller Brown, 2014). However, even though there are no studies on the physiological functions of CaMKIIδC specifically, it likely is the splice isoform that precisely modulates intracellular Ca^2+^ handling in ECC and ETC (Maier and Bers, 2007; Bers, 2011) and the heart rate in fight or flight response (Wu et al., 2009), suggesting that CaMKIIδC is also needed for normal heart function. In addition, the response of cardiomyocytes to the adaptive stimuli Insulin-like growth factor 1, exercise, or the vasoactive peptide alamandine show the dependence of positive effects on Ca^2+^ cycling and contractility on CaMKII activity (Beckendorf et al., 2018; Jesus et al., 2020). Therefore, also with this splice isoform, the question remains when it is detrimental and when it is not. It is hypothesized that spatio-temporal status plays a cardinal role here, and the answer is likely complex. Further research to understand the different functions of CaMKIIδC in different stages of remodeling are needed to fully understand this splice isoform.

## CaMKIIδA

Ca^2+^/calmodulin dependent protein kinase II delta A comprises exon 13 and 15–17 (Figure 2), was first identified as a neuronal CaMKIIδ splice isoform (Schworer et al., 1993), and was only later found in the heart (Xu et al., 2005). The expression of CaMKIIδA is developmentally regulated, with high expression in the neonatal heart (Xu et al., 2005). In the early postnatal period, CaMKIIδA is downregulated, and the adult CaMKIIδB and CaMKIIδC are upregulated. Additionally, CaMKIIδA also is upregulated in hearts from rats with chronic heart failure, in hypoxic cardiomyocytes, and in isoproterenol-treated mice (Li et al., 2011; Gui et al., 2018). CaMKIIδA is preferentially expressed at the T-tubules, in the perinuclear region, and at the intercalated disks (Xu et al., 2005). Unlike CaMKIIδB and CaMKIIδC, the localization of CaMKIIδA only gave limited hints toward its possible functions. However, due to its expression at the *T*-tubules, it has been hypothesized that CaMKIIδA is involved in Ca^2+^ handling by regulating the LTCC. Developmentally, this makes sense, since the developing heart mostly relies on *L*-type calcium current for contraction, and the increased expression of CaMKIIδA in cardiac development supports this line of reasoning (Haddock et al., 1999). However, it must be noted that this hypothesis has not been proven. CaMKIIδA is also implicated in regulating Ca^2+^ handling through regulating RyR2 phosphorylation. In cultured cardiomyocytes, hypoxia increases the expression of CaMKIIδA, which increases RyR2 phosphorylation and SR Ca^2+^ leak, and decreases SERCA2a protein levels (Gui et al., 2018). Also in the case of gene transcription regulation, there is overlap between the different splice isoforms. CaMKIIδA overexpression can, similar to CaMKIIδB and CaMKIIδC, activate the HDAC4-MEF2 axis *in vitro* (Li et al., 2011), and thus induce MEF2-dependent hypertrophy. Whether this effect is reached in a similar way as by the other splice isoforms, or whether this works through a different mechanism, would be interesting to investigate. It is possible that CaMKIIδA, since it can be localized primarily at the *T*-tubules and the perinuclear space, increases transcription through regulating nuclear Ca^2+^. Nuclear and perinuclear Ca^2+^ domains have been described to mediate transcriptional effects (Ibarra et al., 2013), and the involvement of the *T*-tubule associated CaMKIIδA might be an interesting target to study the relation between *T*-tubule signaling and transcription upon Ca^2+^ mobilization.

In the cardiomyocyte specific K.O. of the splicing factor Asf/sf2, CaMKIIδ is misspliced into CaMKIIδA, and this KO mouse presents with Ca^2+^ handling defects, a hypercontractile phenotype, and cardiomyopathy (Xu et al., 2005). To show that increased CaMKIIδA expression is sufficient to induce the Ca^2+^ handling defects seen in the Asf/sf2 KO mice, the authors engineered a mouse model with transgenic overexpression of CaMKIIδA in the heart, and this mouse phenocopied the Asf/sf2-deficient mice (Xu et al., 2005). RBM20 is another splicing regulator that regulates the splicing of CaMKIIδ. Rbm20 KO mice show a shift of CaMKIIδ toward the CaMKIIδA and CaMKIIδ9 splice variants, similar to the Asf/sf2 KO mouse model, and also present Ca^2+^ handling defects, including an increased *L*-type calcium current density (van den Hoogenhof et al., 2018). Overall, these data point toward a causal role for CaMKIIδA in the disturbed Ca^2+^handling in these mouse models. However, unlike the changes seen in hypoxic cardiomyocytes with increased CaMKIIδA expression, in both the Asf/sf2 and Rbm20 KO mouse model, Ryr2 phosphorylation was not increased at the CaMKII-dependent phosphorylation site (Xu et al., 2005; van den Hoogenhof et al., 2018). Therefore, it remains to be investigated how increased CaMKIIδA leads to these Ca^2+^ handling changes. In conclusion, even though some of the downstream effects of CaMKIIδA seem similar to the effects of CaMKIIδC and CaMKIIδB, and there is a significant overlap in function, the phenotype of the transgenic mouse models is clearly different (see Table 2). Therefore, the how, when and where CaMKIIδA is upregulated in the heart, and what the functional consequences are, needs additional attention.

## CaMKIIδ9

The CaMKIIδ9 splice isoform includes exon 16, but not exon 15, in the first variable domain (see Figure 2). It has long been overlooked, but some recent findings have put CaMKIIδ9 onto the radar of CaMKIIδ research. First, a recent paper demonstrated that CaMKIIδ9 is in fact the highest expressed isoform in the human heart, and is also highly expressed in hearts of other mammals such as mice, rabbits, and rhesus monkeys (Zhang et al., 2019). Second, in human RBM20 cardiomyopathy it is CaMKIIδ9 that is upregulated, and not CaMKIIδA, since it seems that CaMKIIδA is not expressed in humans (at least not in adulthood; van den Hoogenhof et al., 2018). CaMKIIδ missplicing is hypothesized to play a major role in the development of DCM caused by RBM20 mutations, and therefore it is important to delineate the functional relevance of CaMKIIδ9 (van den Hoogenhof et al., 2018). The expression of CaMKIIδ9 is upregulated in cultured cardiomyocytes exposed to doxorubicin, oxidative stress, in mice exposed to TAC surgery, and in human heart tissue from dilated cardiomyopathy patients (Zhang et al., 2019). CaMKIIδ9 is localized in the cytoplasm, like CaMKIIδC, and overexpression in cardiomyocytes induces cardiomyocyte cell death through DNA damage and genome instability. This process is mediated by phosphorylation of the ubiquitin E2 enzyme UBE2T, a ubiquitin ligase involved in DNA repair pathways (Machida et al., 2006), which induces its downregulation through proteasomal degradation (Zhang et al., 2019). In the physiological state of the cell, the cytosolic (but not nuclear) UBE2T is phosphorylated by CaMKIIδ9 and thus marked for degradation. Under stress conditions, CaMKIIδ9 expression increases in the heart, which in turn increases the degradation of UBE2T. This disrupts the UBE2T balance between cytosol and nucleus, which negatively affects the repair machinery of DNA and leads to increased DNA damage, genome instability and cell death (Zhang et al., 2019). Transgenic mice overexpressing CaMKIIδ9 present with cardiomyopathy and heart failure, which is attenuated by overexpression of UBE2T (see Table 2). Interestingly, other splice variants of CaMKIIδ do not show regulation of UBE2T signaling, suggesting that although CaMKIIδ9 has almost the same primary structure and cellular localization, it is more pathologically relevant than CaMKIIδA, -δB or -δC in DNA-repair pathways. It would be interesting to further investigate the regulation of other known CaMKIIδ-dependent processes by CaMKIIδ9, such as calcium handling and gene transcription. Overall, these new insights further highlight the importance of splice variant-specific signaling in heart disease.

## CaMKIIδ and Its Splice Variants as Therapeutic Targets

Approximately 518 kinases are encoded in the human genome (Cohen, 2000; Ficarro et al., 2002; Manning et al., 2002), phosphorylating one third of the proteome, so it is not surprising that they are involved in the pathogenesis of various autoimmune, inflammatory, nervous and cardiovascular diseases, and cancer. The treatment of various types of cancer with small kinase inhibitors has been shown to be successful in clinical therapy, but this success has not been achieved in the cardiac field or experimentally tested for CaMKII. In total, the U.S. FDA has approved 52 small molecule protein kinase inhibitors by January 1, 2020, of which 46 are used in the treatment of neoplastic diseases, which shows the rather one-sided success (Roskoski, 2020). Several reasons for this restricted success in cardiac medicine can be outlined, starting with the historically preferred development of ion channel blockers against arrhythmias up to extremely high costs for clinical studies in the cardiovascular field (Fordyce et al., 2015). For CaMKII, the development of specific inhibitors is further complicated by the difference in function but similarity in structure of the different splice isoforms. Nevertheless, CaMKII inhibition has been viewed as a promising therapeutic approach, and multiple studies have shown promising effects. The use of CaMKII inhibitors, such as KN-93, AIP and GS-680, leads to improvement in cardiac function in maladaptive cardiac remodeling animal models and in human heart failure samples (Lebek et al., 2018). However, complete CaMKII inhibition might induce unwanted side effects if it does not specifically target the heart but also many other tissues. In addition, complete CaMKII inhibition affects all downstream pathways, and not only detrimental ones. Therefore, targeting CaMKII more selectively, either by inhibiting specific interactions (e.g., the interaction between CaMKII and a specific phosphorylation target), targeting specific tissues, or by targeting specific CaMKII isoforms, could be of great value. While small molecules and proteins dominate the previous approvals of therapeutics including in the cardiovascular area, the question arises whether these are suitable for CaMKII-directed therapy. Small molecules require a high research effort to achieve the required target specificity and efficiency. Since CaMKII-therapeutics might require an isoform-specific mode of action, the hurdles are even greater because the kinase domains are largely homologous. Protein-based therapeutics, on the other hand, can achieve high specificity, but size, stability and form of administration are challenging (Fosgerau and Hoffmann, 2015). However, the stability *in vivo* is weak due to proteolytic degradation, and their membrane impermeability makes it difficult to find a suitable administration route, as the bioavailable concentration is quickly reduced. While both approaches have been further advanced in recent years, new therapeutic concepts offer potential.

## Antisense Oligonucleotides Targeting CaMKIIδ

One promising method is the use of antisense oligonucleotides (ASOs). ASOs in their original form are short, single-stranded, synthetically produced oligodeoxynucleotides that bind to mRNA targets in a complementary manner and thus modulate mRNA and protein expression (Dias and Stein, 2002). The first “naked” ASOs induced toxicity, and had an insufficient specificity as well as low biological activity (Rinaldi and Wood, 2018). In the last years, the design of RNA therapeutics has made significant advances leading to new generations of chemically modified ASOs. While early ASOs were only active via endonuclease (RNase H) mediated mRNA degradation (Eder et al., 1991), new ASOs can, for example, sterically block splicing factors or prevent ribosome recruitment to inhibit translation (Muntoni and Wood, 2011). This makes the use of ASOs a promising approach to not only decrease expression of targets, but also to redirect splicing. The first ASO therapy was approved by the FDA for the treatment of chorioretinitis, an inflammation of the retina caused by the cytomegalovirus (Roehr, 1998). The method has also successfully been tested in other diseases, such as Duchenne muscular dystrophy (Goemans et al., 2011), spinal muscular atrophy (Hua et al., 2011), and myotonic dystrophy (Wheeler et al., 2012). For the treatment of Duchenne muscular dystrophy, the therapeutic agent Eteplirsen was developed and approved by FDA in 2016, which promotes the production of a shortened, but active form of the dystrophin protein (Mendell et al., 2013). In theory, RNAi-based therapeutics are suitable for a CaMKII-directed treatment. Both systems, the knockdown of detrimental isoforms by mRNA degradation as well as targeting specific splice sites to redirect splicing could lead to success. Since loss of CaMKIIδ improves cardiac function, an RNAi-mediated knockdown of the entire gene would be the straightforward attempt for a therapy. In RBM20 cardiomyopathy, the overall level of CaMKIIδ is increased and at the same time CaMKIIδ splicing shifts toward CaMKIIδA and CaMKIIδ9. An RNAi-mediated knockdown of CaMKIIδA and CaMKIIδ9, e.g., by targeting exon 16, would be an appropriate approach. CaMKIIδ KO mice are protected from cardiac dysfunction in an ischemia/reperfusion injury model, with additional protective effects in case of parallel overexpression of CaMKIIδB. For patients suffering from acute myocardial infarction, RNAi therapeutics could be developed to redirect splicing in favor of CaMKIIδB, e.g., by blocking corresponding splice sites/factors. Alternatively, expressing exogenous CaMKIIδB through the use of gene therapy could have a protective effect and reduce the extent of dysfunction.

Probably the most important problem, however, is the delivery *in vivo*, since ASOs circulate in the bloodstream after intravenous or subcutaneous injection and can accumulate in the liver, kidney or spleen (Roberts et al., 2020) and may also be toxic. In addition, ASOs are large, hydrophilic polyanions that cannot easily cross the plasma membrane and must resist a variety of mechanisms, such as nuclease degradation in the extracellular space (Tsui et al., 2002) or removal by renal clearance (Iversen et al., 2013). Finally, it is currently not possible to direct ASOs to specific tissues such as the heart, but treatment is rather systemic. Due to these hurdles, it is not surprising that most approved RNAi therapeutics to date are limited to local administration (e.g., pegaptanib in the eye) or targeting the liver (due to the discontinuous sinusoidal endothelium, which allows for easy uptake of ASOs). Therefore, alternative and more advanced delivery systems have been developed in recent years and the pharmacokinetic properties of RNAi therapeutics have been improved by chemical modifications. Spherical nucleic acids (Kapadia et al., 2018), exosome loading, and nanotechnological systems or “intelligent materials” are promising approaches (Roberts et al., 2020). Despite all innovations, it remains unclear whether the progress made in RNAi technology to date is sufficient to develop a successful CaMKIIδ-directed therapy. Apart from cardiomyocytes, CaMKIIδ is also the dominant isoform in smooth muscle cells (House et al., 2007) and endothelial cells (Wang et al., 2010; McCluskey et al., 2019; Dalal et al., 2021), so differences in cellular function need to be investigated to predict and avoid potential side effects. In general, it is important to further investigate the precise role of the different splice isoforms of CaMKIIδ, because the complex and diverse involvement of CaMKIIδ in various cellular and intracellular processes will require precise fine-tuning of therapeutics. In addition, CaMKII-directed therapeutics should consider the spatio-temporal distribution of the various splice isoforms. RNAi based therapeutics look more promising due to their high specificity, but the problem of cardiac-specific delivery needs further investigation and new solutions.

Overall, CaMKIIδ-directed therapeutics show great promise for three main reasons: (1) its levels are upregulated in both experimental models of heart failure and human samples which suggests a causal role for increased CaMKIIδ activation in disease, (2) the transgenic overexpression of CaMKIIδA, -δC and -δ9 leads to adverse cardiac remodeling accompanied by contractile dysfunction, and most importantly (3) CaMKIIδ inhibition is generally associated with protection against cardiac dysfunction and a reduction in cardiac damage in animal models subjected to detrimental stimuli. Nevertheless, the importance of CaMKIIδ in physiological processes, as well as the proposed protective effects of, e.g., CaMKIIδB suggest that CaMKIIδ-directed therapeutics require high specificity [For further details see (Nassal et al., 2020)].

## Conclusion and Future Directions

The first report of the role of Ca^2+^ in the heart came from work of Ringer in the nineteenth century who accidentally used tap water instead of distilled water in his NaCl solution, and then found out that Ca^2+^ was necessary for contraction (Ringer, 1883; Eisner, 2014). Since then, Ca^2+^ has been demonstrated to be crucial in numerous processes in the heart, one of which being Ca^2+^-dependent signaling. CaMKII is one of the major transducers of specific spatio-temporal Ca^2+^ signaling, and has been the focus of many (cardiac) studies for decades (Clapham, 2007). These studies have led to tremendous insight into CaMKII-dependent signaling, but also to many new questions. One basic question that remains, is the function of the holoenzyme and how this is affected by the different splice variants. The dodecameric holoenzyme formation of CaMKII is distinct from structures of other protein kinases (Rosenberg et al., 2005). Based on the evolutionary conservation of this feature, it is thought that oligomerization plays a central role in the functioning of CaMKII. Phosphorylation spreading (autophosphorylation) within the holoenzyme and the exchange of activated and inactivated subunits between holoenzyme formations (Stratton et al., 2014) are only two special features offered by this structural setup. Investigating the structural features of CaMKII, and specifically CaMKIIδ, is essential for accurately addressing future therapeutics, and the comprehensive structure-function links have yet to be adequately discovered. However, modern technologies, such as molecular dynamics, offer novel tools and initial experiments have indicated, for example, that regulatory segments can spontaneously dock to the interfaces between hub subunits or that the length of the linker region controls the balance between activating or inhibitory autophosphorylation (Bhattacharyya et al., 2020; Karandur et al., 2020). It would be interesting to study how the dynamics of the enzyme structure changes during different periods in the cell, for example, during changing Ca^2+^/CaM concentrations between Ca^2+^ pulse events. In addition, different splice isoform ratios within the oligomer and their effect on the structure and dynamics are not fully understood and should be investigated. In addition, the functional difference of the different splice isoforms of CaMKIIδ, and how to exploit these differences, is only partly understood. In line with this, the differential effects of the CaMKIIδ isoforms could be related with its interactome. Hence, unraveling of their possible interaction partners (such as PKA) could clarify why these isoforms exert different and/or redundant functions. Moreover, the 4 isoforms described in this review are not the only CaMKIIδ splice isoforms expressed in the heart. Even though less highly expressed, other splice isoforms such as CaMKIIδ4 are also found in the heart, and the function and relevance of these isoforms has not been explored. The fact that CaMKIIδ9, after being overlooked for a long time, turns out to be one of the highest expressed isoforms with a relevant function in the heart, illustrates that lesser known isoforms should not be neglected (Zhang et al., 2019). Another interesting question relates to the spatio-temporal control of activation of (splice variants of) CaMKIIδ. For example, CaMKIIδ’s early response to detrimental stimuli is compensatory, but turns maladaptive in later stages. A more specific approach to CaMKIIδ inhibition, be it through targeting single splice isoforms, focusing on specific interactions, and on specific stages of CaMKIIδ activation will therefore be extremely valuable. In conclusion, tremendous progress has been made in our understanding of CaMKIIδ in the heart, but the picture is not yet complete. More specific studies will likely lead to new insights into this multifunctional and versatile protein, and could yield new therapeutic possibilities.

## Author Contributions

All authors contributed to writing and revising the manuscript.

## Conflict of Interest

The authors declare that the research was conducted in the absence of any commercial or financial relationships that could be construed as a potential conflict of interest.
